# Supplementary material for: Overexpression of blaOXA-58 Gene Driven by ISAba3 Is Associated with Imipenem Resistance in a Clinical Acinetobacter baumannii Isolate from Vietnam
Source: Biomed Res Int. 2020 Jul 31;2020:7213429. doi: 10.1155/2020/7213429 (PMC7420922; doi:10.1155/2020/7213429)
Supplement: Supplementary Materials — Figure S1: result of imipenem E-test for five clinical isolates of A. baumannii (blaOXA-58). Figure S2: electrophoresis results of PCR screening for the presence/absence of ISAba1, ISAba2, ISAba3, ISAba4, and IS18 in five clinical isolates of A. baumannii (blaOXA-58). Figure S3: electrophoresis results of PCR for the presence/absence of insertion sequence (IS) upstream of blaOXA-58 gene. Figure S4: duplex real-time RT-PCR analysis of the blaOXA-51 and blaOXA-58 mRNA relative expression compared with 16S rRNA in five A. baumannii isolates. Figure S5: Bradford assay standard curve of concentration versus absorbance for protein quantification. Figure S6: nitrocefin standard curve. Table S1: duplex real-time RT-PCR analysis of the blaOXA-51 and blaOXA-58 mRNA relative expression in three A. baumannii isolates under conditions with oxacillin as an inducer or without oxacillin induction. Table S2: results for protein quantification of supernatant and periplasmic fractions. Table S3: results for β-lactamase activity of supernatant and periplasmic fractions. [file 7213429.f1.pdf]

Overexpression of *bla*$_{OXA-58}$ Gene Driven by IS*Aba3* is Associated with Imipenem Resistance in a Clinical *Acinetobacter baumannii* Isolate from Vietnam

Anh T. Nguyen, Son C. Pham, Anh K. Ly, Chau V.V. Nguyen, Thanh T. Vu, Tuan M. Ha

Supplementary Material

Tables of contents

- Figure S1. Result of imipenem E-test for five clinical isolates of *A. baumannii* (*bla*$_{OXA-58}$)
- Figure S2. Electrophoresis results of PCR screening for the presence / absence of IS*Aba1*, IS*Aba2*, IS*Aba3*, IS*Aba4*, and IS18 in five clinical isolates of *A. baumannii* (*bla*$_{OXA-58}$)
- Figure S3. Electrophoresis results of PCR for the presence / absence of insertion sequence (IS) upstream of *bla*$_{OXA-58}$ gene.
- Figure S4. Duplex real-time RT-PCR analysis of the *bla*$_{OXA-51}$ and *bla*$_{OXA-58}$ mRNA relative expression compared with 16S rRNA in five *A. baumannii* isolates.
- Figure S5. Bradford assay standard curve of concentration versus absorbance for protein quantification.
- Figure S6. Nitrocefin Standard Curve.
- Table S1. Duplex real-time RT-PCR analysis of the *bla*$_{OXA-51}$ and *bla*$_{OXA-58}$ mRNA relative expression in three *A. baumannii* isolates under conditions with oxacillin as an inducer or without oxacillin induction.
- Table S2. Results for protein quantification of supernatant and periplasmic fractions.
- Table S3. Results for β-lactamase activity of supernatant and periplasmic fractions.

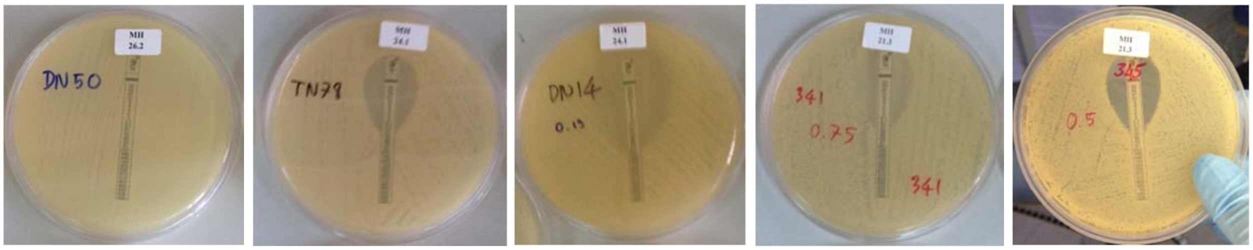

Figure S1. Result of imipenem E-test for five clinical isolates of *A. baumannii* (*bla*$_{\text{OXA-58}}$)

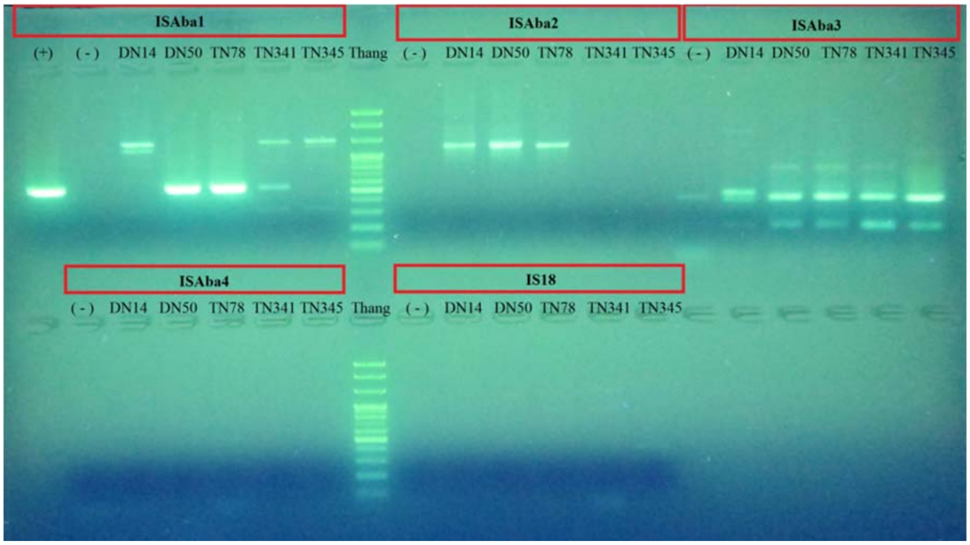

Figure S2. Electrophoresis results of PCR screening for the presence / absence of IS*Aba1*, IS*Aba2*, IS*Aba3*, IS*Aba4*, and IS18 in five clinical isolates of *A. baumannii* (*bla*$_{\text{OXA-58}}$)

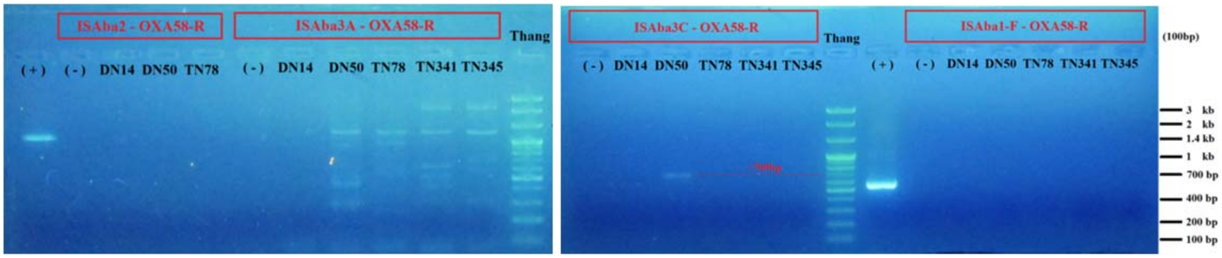

Figure S3. Electrophoresis results of PCR for the presence / absence of insertion sequence (IS) upstream of *bla*$_{\text{OXA-58}}$ gene.

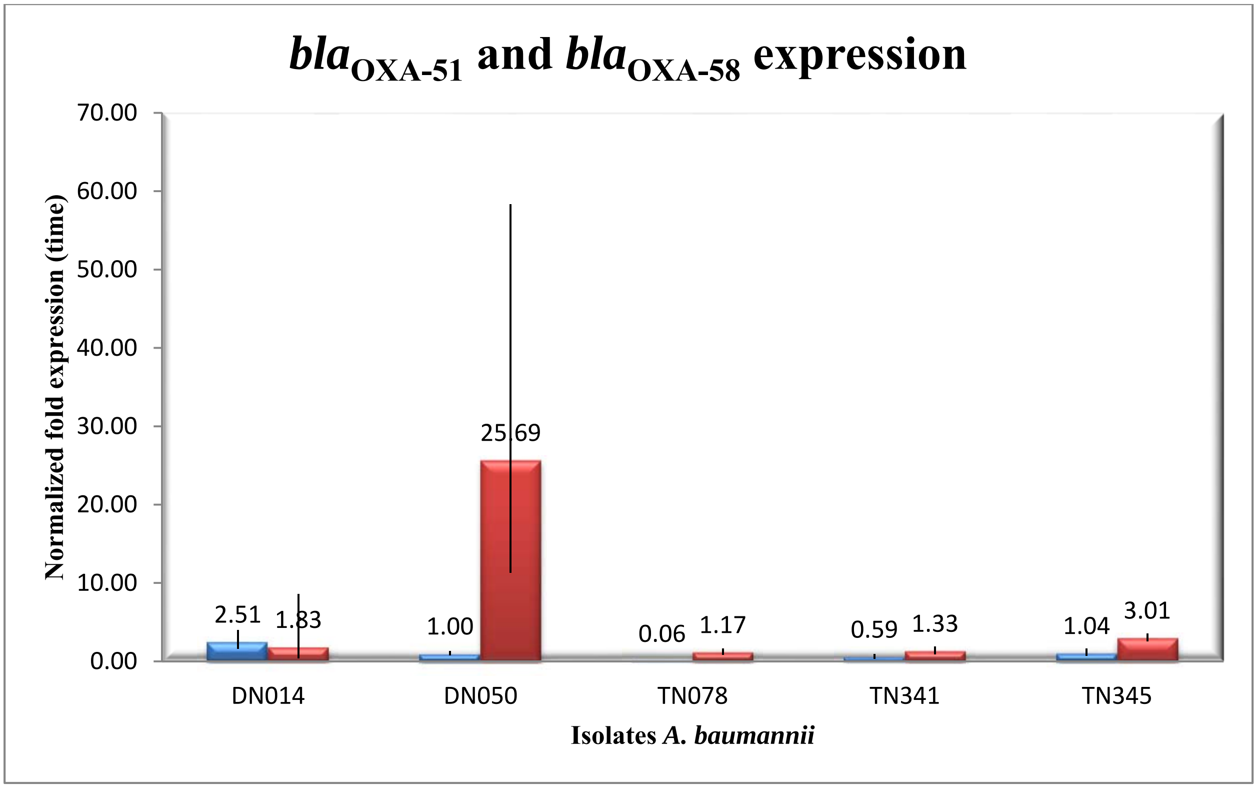

Figure S4. Duplex real-time RT-PCR analysis of the $bla_{\text{OXA-51}}$ and $bla_{\text{OXA-58}}$ mRNA relative expression normalized to 16S rRNA gene as a reference in five *A. baumannii* isolates. The expression of $bla_{\text{OXA-51}}$ in isolate DN050 is used as a calibrator. Error bars represent deviation for the normalized fold expressions of $bla_{\text{OXA-51}}$ and $bla_{\text{OXA-58}}$ in five isolates induced by oxacillin. Blue and red colours represent the expression of $bla_{\text{OXA-51}}$ and $bla_{\text{OXA-58}}$ correspondingly.

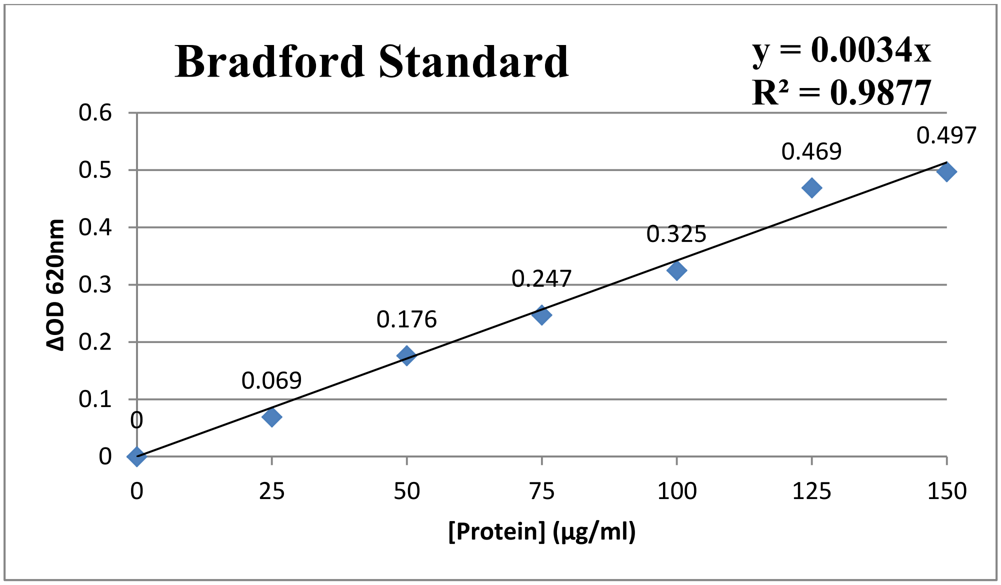

Figure S5. Bradford assay standard curve of concentration versus absorbance for protein quantification.

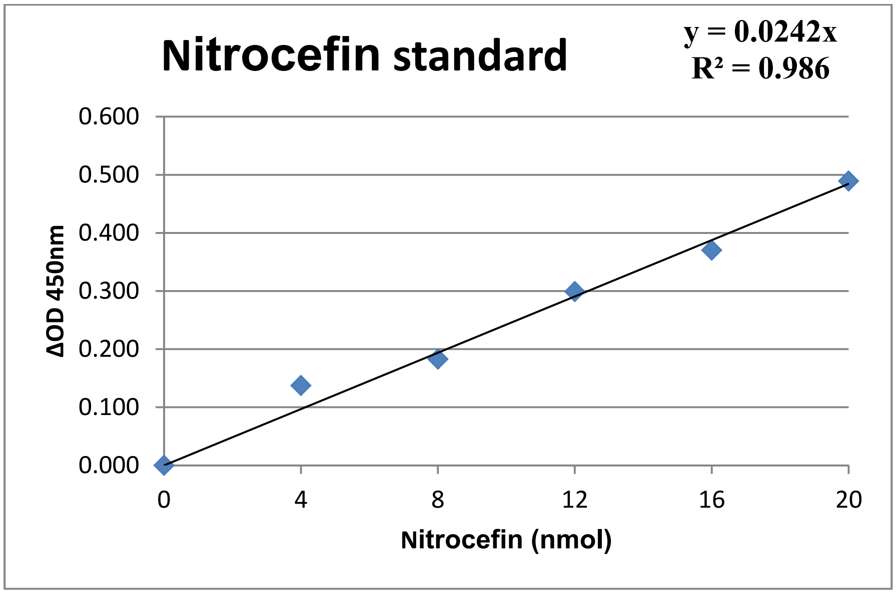

Figure S6. Nitrocefin Standard Curve

Table S1. Duplex real-time RT-PCR analysis of the *bla*$_{OXA-51}$ and *bla*$_{OXA-58}$ mRNA relative expression in three *A. baumannii* isolates under conditions with oxacillin as an inducer or without oxacillin induction. 16S rRNA gene is used as the reference for normalization and the non-induced control is run as the calibrator.

**Isolate**		**DN050**			**TN341**			**TN345**		
		Min	Value	Max	Min	Value	Max	Min	Value	Max
Relative expression of *bla*$_{OXA-51}$ (time)	Not induced	0.4	1.0	2.3	1.0	1.0	1.0	0.9	1.0	1.1
	Induced 1	1.9	4.60	11.2	0.5	0.9	1.5	0.1	0.2	0.4
	Induced 2	1.8	3.10	5.6	0.3	0.7	1.6	0.8	1.2	2.0
	Induced 3	6.5	7.0	7.6	-	-	-	-	-	-
Relative expression of *bla*$_{OXA-58}$ (time)	Not induced	0.7	1.0	1.4	0.8	1.0	1.2	0.7	1.0	1.5
	Induced 1	10.7	14.4	19.3	0.3	0.7	1.5	0.3	0.5	0.9
	Induced 2	15.9	22.3	31.3	0.3	0.8	2.0	0.5	0.6	0.7
	Induced 3	16.6	20.1	24.4	-	-	-	-	-	-

Table S2. Results for protein quantification of supernatant and periplasmic fractions. The concentration of protein (µg/ml) was determined using the equation y = 0.0034x with an R^2 value of 0.9877, where y is absorbance and x is concentration.

**Isolate**	**DN050**	**TN078**	**DN014**	**TN341**	**TN345**
Supernatant (µg/ml)	58.82	45.29	37.06	47.94	45.88
Periplasmic (µg/ml)	22.94	85.00	26.47	18.24	37.35

Table S3. Results for β-lactamase activity of supernatant and periplasmic fractions. The hydrolyzed Nitrocefin (nmol) generated by β-lactamase during the reaction time (ΔT) was determined using the equation y = 0.0242x with an R^2 value of 0.986, where y is absorbance and x is the amount of hydrolyzed Nitrocefin. The β-lactamase activity of the test samples is calculated based on the formula: B/(ΔTxV)xD (nmol/min/ml) or (mU/ml) where B is the amount of Nitrocefin from the Standard Curve (nmol), ΔT is the reaction time (min), V is the sample volume added into the reaction well (ml), D is the sample dilution factor.

**Sample**	**Isolate**	**ΔOD**	**ΔT (min)**	**B (nmol)**	**V (ml)**	**Diluted (D)**	**β-lactamase activity (mU/ml)**	**(mU/µg)**	**SD**
**Supernatant**	DN050	0.385	25	15.9	0.005	5	636.4	10.8	3.3
	TN078	0.388	20	16.0	0.005	5	801.7	17.7	5.2
	DN014	0.299	24	12.4	0.005	5	514.8	13.9	4.1
	TN341	0.188	15	7.8	0.005	5	517.9	10.8	3.3
	TN345	0.201	14	8.3	0.005	5	593.3	12.9	3.8
**Periplasmic**	DN050	0.310	25	12.8	0.005	10	1024.8	44.7	12.8
	TN078	0.251	16	10.4	0.005	10	1296.5	15.3	4.3
	DN014	0.219	24	9.0	0.005	10	754.1	28.5	8.2
	TN341	0.197	18	8.1	0.005	10	904.5	49.6	16.0
	TN345	0.124	10	5.1	0.005	10	1024.8	27.4	10.1
